# Predicting expression: the complementary power of histone modification and transcription factor binding data

**DOI:** 10.1186/1756-8935-7-36

**Published:** 2014-11-24

**Authors:** David M Budden, Daniel G Hurley, Joseph Cursons, John F Markham, Melissa J Davis, Edmund J Crampin

**Affiliations:** Systems Biology Laboratory, Melbourne School of Engineering, The University of Melbourne, 3010 Parkville, Australia; NICTA Victoria Research Laboratory, The University of Melbourne, 3010 Parkville, Australia; The Walter and Eliza Hall Institute of Medical Research, Department of Medical Biology, The University of Melbourne, 3010 Parkville, Australia; ARC Centre of Excellence in Convergent Bio-Nano Science and Technology, 3010 Parkville, Australia; Department of Mathematics and Statistics, The University of Melbourne, 3010 Parkville, Australia; School of Medicine, The University of Melbourne, 3010 Parkville, Australia

**Keywords:** Gene expression, Transcriptional regulation, Transcription factors, Histone modifications, Predictive modelling

## Abstract

**Background:**

Transcription factors (TFs) and histone modifications (HMs) play critical roles in gene expression by regulating mRNA transcription. Modelling frameworks have been developed to integrate high-throughput omics data, with the aim of elucidating the regulatory logic that results from the interactions of DNA, TFs and HMs. These models have yielded an unexpected and poorly understood result: that TFs and HMs are statistically redundant in explaining mRNA transcript abundance at a genome-wide level.

**Results:**

We constructed predictive models of gene expression by integrating RNA-sequencing, TF and HM chromatin immunoprecipitation sequencing and DNase I hypersensitivity data for two mammalian cell types. All models identified genome-wide statistical redundancy both within and between TFs and HMs, as previously reported. To investigate potential explanations, groups of genes were constructed for ontology-classified biological processes. Predictive models were constructed for each process to explore the distribution of statistical redundancy. We found significant variation in the predictive capacity of TFs and HMs across these processes and demonstrated the predictive power of HMs to be inversely proportional to process enrichment for housekeeping genes.

**Conclusions:**

It is well established that the roles played by TFs and HMs are not functionally redundant. Instead, we attribute the statistical redundancy reported in this and previous genome-wide modelling studies to the heterogeneous distribution of HMs across chromatin domains. Furthermore, we conclude that statistical redundancy between individual TFs can be readily explained by nucleosome-mediated cooperative binding. This could possibly help the cell confer regulatory robustness by rejecting signalling noise and allowing control via multiple pathways.

**Electronic supplementary material:**

The online version of this article (doi:10.1186/1756-8935-7-36) contains supplementary material, which is available to authorized users.

## Background

A critical challenge in molecular biology is understanding the biological mechanisms underlying precise spatiotemporal regulation of gene expression in mammals. Significant regulation is known to occur at the level of transcriptional initiation and elongation [1], through the combinatorial interactions of transcription factors (TFs) [2, 3] and histone modifications (HMs) [4, 5]. By binding to specific DNA motifs, activator or repressor TFs regulate the recruitment and behaviour of RNA polymerase II (RNAP-II). Direct interactions between TFs and the transcription pre-initiation complex require genomic proximity to the transcription start site (TSS) or higher-order chromatin looping [6], corresponding with TF-binding motifs in the promoter or enhancer/silencer regions respectively [2, 7]. Post-translational modifications of the amino-termini of nucleosomal histones are also known to regulate transcription [8, 9] by either modulating the local chromatin structure to control TF accessibility [4] or directly recruiting chromatin remodellers and other related enzymes [10]. Altered gene expression caused by abnormalities in TF or HM patterns has been directly associated with hundreds of human diseases [3], including leukaemia [11], prostate cancer [12] and various developmental, autoimmune, neurological, inflammatory and neoplastic disorders [13].

The complex relationship between TFs and HMs is still largely unexplored. Statistical models have recently been developed to integrate high-throughput omics data with the aim of understanding the regulatory logic that follows from these interactions (recently reviewed in [14]). These models demonstrated that TFs and HMs are accurate predictors of mRNA transcript abundance in several organisms and cell types. However, the utility of this data-driven framework is not the ability to predict gene expression, but rather the insights that can be gained from investigating the putative regulatory interactions captured by an accurate model. A recent study showed that models constructed from position weight matrix-predicted TF binding, when combined with a tissue-specific H3K4me3 prior, yield similar prediction accuracy to models constructed from actual chromatin immunoprecipitation sequencing (ChIP-seq) data [15]. Furthermore, a principal component analysis of these models was able to extract correctly the established regulatory roles (i.e., activator or repressor) of 20 TFs and HMs in mouse embryonic stem cells (mESCs) [14].

In addition to providing a powerful explorative framework, predictive modelling of gene expression has yielded an unexpected and previously unexplained result: that TFs and HMs are statistically redundant in explaining mRNA transcript abundance at a genome-wide level. Moreover, redundancy has been identified both *within* and *between* TFs and HMs in mESCs [16], to the extent that a single TF (E2f1) is almost as informative as a panel of 20 TFs and HMs with well-established regulatory roles [15]. Here, *statistical* redundancy equates to two variables providing equivalent information (e.g., due to being strongly correlated), and it is important to appreciate that this does not necessarily imply *functional* redundancy (i.e., removing either element does not affect gene expression). Assuming the existence of functional redundancy between TFs and HMs outwardly contradicts our understanding of transcriptional regulation, in which TFs and HMs play complementary yet distinct roles in RNAP-II recruitment and elongation.

In this study, we investigate the fundamental cause of the statistical redundancy within and between TFs and HMs. First, we validate the robustness of previous findings by constructing genome-wide predictive models for different mammalian cell types and modelling algorithms. We confirm that TFs and HMs are both predictive of gene expression (measured by mRNA transcript abundance) and statistically redundant at a genome-wide level. Our analysis was extended by constructing individual models for thousands of ontology-classified biological processes. By diverging from previous genome-wide analyses, we identify significant variance in the distribution of statistical redundancy across these processes, which we attribute to regions of open nucleosome-sparse chromatin maintained by the activity of boundary proteins and enriched for housekeeping genes. Finally, we discuss several implications of our findings and how they contribute to the overall understanding of regulatory logic in mammalian systems.

## Results and discussion

### Transcription factors and histone modifications are predictive of mRNA transcript abundance

As TFs and HMs are known to play critical roles in regulating transcription, accurate predictive models of mRNA transcript abundance have been constructed from corresponding ChIP-seq binding data for various organisms, cell types and modelling techniques [14, 17–19]. To validate the robustness of these findings, we constructed both log-linear and support vector regression (SVR) models for two mammalian cell types: mESCs and human lymphoblastoids (GM12878).

Table 1 presents the prediction accuracy of log-linear and SVR models constructed from three sets of data: TF binding (TF), HM and DNase-I hypersensitivity (HM+DNase; both proxies for chromatin accessibility) and the concatenation of both (TF+HM+DNase). The proportion of transcript abundance variation explained by each model (adjusted *R*^2^) was calculated using a tenfold cross-validation [20], with the presented adjusted *R*^2^ values capturing the mean and standard deviation of these folds. The relationship between measured and predicted mRNA transcript abundance is visualised in Additional file 1: Figure S1 and Additional file 2: Figure S2 for mESCs and GM12878 cells, respectively.

**Table 1:** **Prediction accuracy of predictive models of mRNA transcript abundance**

	TF	HM+DNase	TF+HM+DNase
**mESC**			
Log-linear regression	0.58 (0.01)	0.62 (0.01)	0.68 (0.01)
Support vector regression	0.64 (0.02)	0.67 (0.01)	0.70 (0.01)
**GM12878**			
Log-linear regression	0.33 (0.01)	0.42 (0.01)	0.43 (0.01)
Support vector regression	0.39 (0.01)	0.45 (0.01)	0.46 (0.01)

Three sets of ChIP-seq input data were considered: TF binding (TF), HM and DNase-I hypersensitivity (HM+DNase) and the concatenation of both (TF+HM+DNase). Prediction accuracy is based on tenfold cross-validation adjusted R^2^, reported as the mean and standard deviation of the ten folds.

ChIP, chromatin immunoprecipitation; HM, histone modification; mESC, mouse embryonic stem cell; seq, sequencing; TF, transcription factor.

It is evident that small sets of TFs, HMs and DNase are predictive of genome-wide mRNA transcript abundance in mESCs, as reported in previous studies [18]. We have further demonstrated that these results extend to different mammalian cell types and are robust against algorithm selection. As support vector regression (SVR) yields minimal improvement despite a two-order-of-magnitude increase in required CPU time, only log-linear regression is applied throughout the remainder of this study.

### Transcription factors and histone modifications are less predictive of mRNA transcript abundance in differentiated cells

Our results reveal that models of transcriptional regulation in GM12878 cells are less accurate than those constructed for mESCs. To ensure that this does not simply reflect the different proportion of zero-expression genes in the GM12878 and mESC data, we constructed log-linear regression models considering only genes with R/FPKM(fragments per kilobase per million)-normalised transcript abundance >0. These models yielded an average reduction in adjusted *R*^2^ prediction accuracy of 58% and 7% for GM12878 and mESC, respectively (not shown), excluding a high proportion of zero-expression genes in the differentiated GM12878 cell line as the underlying cause of the observed performance gap. The removal of these genes also adversely affected subsequent analysis (not shown), as much of the information used to elucidate the silencing roles of some regulatory elements (e.g., H3K9me3 and H3K27me3) is lost.

One explanation for the performance gap between mESC and GM12878 models may be the selection of individual TFs, which vary between cell types. The 12 TFs selected for mESCs (see Table 2) are known to play important regulatory roles specific to embryonic stem cell biology; i.e., as self-renewal regulators and pluripotency reprogramming factors [21, 22]. Initial differentiation of embryonic stem cells involves silencing of these TFs and activation of developmental regulators [23, 24], necessitating the selection of alternate TFs for GM12878 modelling. Although the 11 GM12878 TFs chosen are known to play key roles (see Table 2) in regulating various cellular, metabolic and development processes [3], it is possible that they represent a smaller fraction of the key regulators than the 12 considered for mESCs (in which regulatory logic is better characterised).

**Table 2:** ***Mus musculus*** **(embryonic stem cell) data**

Data type	Data source	Notes
RNA-seq	[25]	18,936 genes RPKM-normalised [26]
TSS	Ensembl mm8/NCBIM36.46 [27]	Consider only most 5 ^′^-located TSS for each gene
TF ChIP-seq	[21, 28]	E2f1, Esrrb, Klf4, c-Myc, n-Myc, Nanog, Oct4, Smad1, Sox2, Stat3, Tcfcp2l1 and Zfx
HM ChIP-seq	[29–31]	H3K4me1, H3K4me2, H3K4me3, H3K9me3, H4K20me3, H3K27me3 and H3K36me3
DNase-seq	[15, 32]	DNase I hypersensitivity
Gene ontology	[33, 34]	GOC validation date: 15 November 2013Structure from GO.db R package
Housekeeping annotations	[35]	3,689 orthologs inferred from the MGI human-mouse homology

Genes corresponding with haplotype variants, unmapped contig regions and low confidence RNA-seq mappings were removed, resulting in a set of 17,517 genes for analysis. Pre-processed RNA-seq and ChIP-seq data and mapped DNase I hypersensitivity in mESCs are available online [15, 32].

ChIP, chromatin immunoprecipitation; GOC, Gene Ontology Consortium; HM, histone modification; MGI, Mouse Genome Informatics; seq, sequencing; TF, transcription factor; TSS, transcription start site; RPKM, reads per kilobase per million.

A more likely explanation for the performance gap between predictive models for embryonic stem versus differentiated cells is the increasing heterogeneity in regulatory mechanisms following differentiation. In embryonic stem cells, the majority of gene promoters containing CpG (dinucleotide) islands are characterised by H3K4me3-bearing nucleosomes. Many of these genes are maintained in a bivalent state with the inherently antagonistic H3K27me3 repressive mark; these genes are expressed only at low levels, but are poised for rapid transition to active or silenced states in response to differentiation signals or other extracellular stimuli [36]. Accordingly, the genome-wide correlation between H3K4me3 and H3K27me3 in mESCs is very high (Pearson’s *r* = 0.78 versus -0.20 in GM12878, not shown).

Lineage-specific gene expression programs exhibit far less regulatory homogeneity; e.g., many genes are silenced by H3K27me3/polycomb-mediated facultative heterochromatinisation, whereas others are silenced by H3K9me3/HP1-mediated constitutive heterochromatinisation and subsequent DNA methylation. Synergistic and conditional relationships become more widespread (e.g., H3K4me3 is positively associated with expression only in the absence of H3K27me3), limiting the effectiveness of regression models only able to capture additive and simple-multiplicative relationships.

Future predictive modelling studies of differentiated cells could integrate information for the H2A.Z histone variant (not assayed), which is critical for maintaining metastable equilibrium between antagonistic H3K4me3 and H3K27me3 [37, 38], and could therefore be used to classify genes subject to different regulatory logic.

### Individual transcription factors are statistically redundant for predicting mRNA transcript abundance

**Figure 1:**
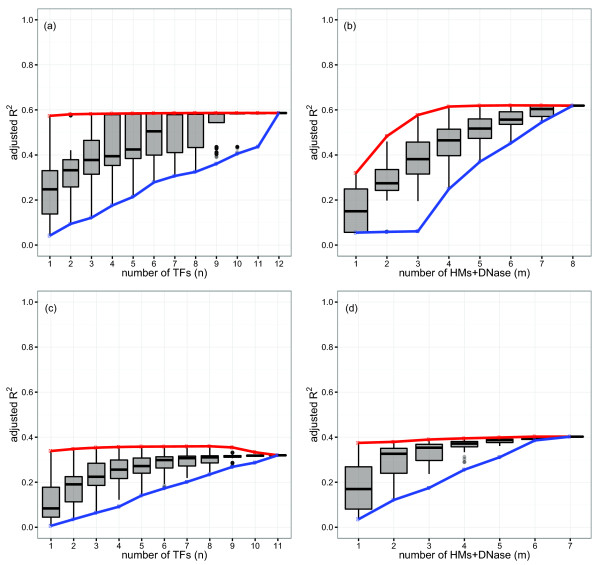
**Statistical redundancy within TFs and HMs in predicting genome-wide mRNA transcript abundance.** **(a,b)** mESCs and **(c,d)** GM12878 cells. Adjusted *R* ^2^ distributions of the log-linear regression models for all combinations of *n* TFs **(a,c)** and *m* HMs and DNase **(b,d)**. The minimum and maximum prediction accuracies for each *n* and *m* are connected by the blue and red curves, respectively. Although models constructed from more regulatory elements generally yielded improved prediction accuracy, the rapidly diminishing improvement when adding additional elements to the model suggests significant statistical redundancy within TFs and HMs. It is important to note that statistical redundancy does not necessarily imply functional redundancy. HM, histone modification; TF, transcription factor.

Functional redundancy between individual TFs has previously been observed in *Saccharomyces cerevisiae* [5] and *Drosophila melanogaster* [19] and proposed as an important mechanism in eukaryotes [2]. To investigate the existence of similar redundancy within mammalian TFs and HMs, log-linear regression models of genome-wide mRNA transcript abundance were constructed for all combinations of *n* TFs and *m* HMs and DNase considered in this study. Figure 1(a,b) demonstrates the adjusted *R*^2^ distributions for these 4,095 mESC TF models and 255 mESC HM+DNase models respectively, with the minimum and maximum prediction accuracies for each *n* and *m* connected by the blue and red curves. The corresponding results for GM12878 models are presented in Figure 1(c,d).

Although models constructed from greater numbers of regulatory elements generally yielded improved prediction accuracy, this improvement diminished considerably for values of *n* or *m* greater than 4. This is particularly evident for mESC TF models; despite all 12 TFs being known to play key roles in mESC biology [21], a model constructed from E2f1 data alone yielded equivalent mRNA transcript abundance prediction accuracy (adjusted *R*^2^ = 0.57) as a model integrating all 12 (adjusted *R*^2^ = 0.59). The most predictive TFs (E2f1, c-Myc, n-Myc and Zfx) were those known to localise preferentially to promoter regions (e.g., E2f1 was found to bind to more than 60% of mESC promoters), whereas the least predictive TFs (Smad1 and Stat3) bind further from the TSS (e.g., Smad1 was found to bind to less than 3% of mESC promoters).

Previous studies have demonstrated that many TFs (including E2f1 and Oct4) do not require the presence of a consensus motif to bind *in vivo*, but rather may be recruited to the promoter with the assistance of other bound TFs [39, 40]. These results may partially explain the observed statistical redundancy between mammalian TFs; i.e., if one TF is necessary for the recruitment of another, these TFs will provide similar predictive information regarding the regulatory state despite their distinct functional roles. It should be noted that this is distinct from *functional* redundancy; i.e., it does not imply the removal of any individual TF would not affect gene expression. It has previously been proposed that the parallel deployment of cooperatively bound TFs confers robustness to gene expression, both allowing the regulatory state of a gene to reject signalling noise and providing control through activation or inhibition of multiple signalling pathways [41].

### Transcription factors and histone modifications provide equivalent information regarding genome-wide transcriptional regulation

In Table 1, we present the prediction accuracy of log-linear and SVR models constructed from three sets of data: TF binding (TF), HM and DNase-I hypersensitivity (HM+DNase) and the concatenation of both (TF+HM+DNase). In addition to the findings described earlier, it is apparent that the TF+HM+DNase models perform only marginally better than those constructed from TF or HM+DNase data alone, irrespective of algorithm or cell type. Although TFs and HMs independently provide significant information regarding transcriptional regulation, it appears that they provide the *same* information and are therefore statistically redundant. To quantify this phenomenon, we performed a partial correlation analysis by calculating the correlation between genome-wide transcript abundance prediction residuals for TF and HM+DNase models [42]. The residuals were found to be highly correlated (Pearson’s *r* > 0.8 for both mESCs and GM12878 cells, not shown), indicating a significant degree of association between the TF and HM+DNase data. These results (previously identified for mESCs [15, 16]) outwardly contradict our understanding of transcriptional regulation, in which TFs and HMs play complementary yet distinct roles in RNAP-II recruitment and elongation.

### Prediction of transcript abundance for genes grouped by biological process suggests a more heterogeneous role for transcription factors and histone modifications

To investigate the source of statistical redundancy between TFs and HMs, we constructed individual predictive models for thousands of ontology-classified biological processes. Insight can be gained into the nature of this redundancy by investigating its distribution across the smaller groups of genes contributing to each process.

The mESC and GM12878 genes were grouped by ontology-classified biological process. Two regression models were constructed for each set of genes: one considering only TF-binding data and the other considering only HM and DNase data. The ratio of adjusted *R*^2^ values for the TF and HM+DNase models was calculated to capture their relative performance.

Of the 1,880 mESC processes considered, 25 were found to exhibit a significant TF-to-HM+DNase adjusted *R*^2^ ratio (i.e., demonstrating that TF binding is more predictive of mESC mRNA transcript abundance than HMs and DNase for the markers considered in this study, Benjamini–Hochberg-corrected *P* < 0.05 [43]). A full list of these processes and their respective ratios is provided in Additional file 3: Table S1. Furthermore, 523 processes were found to exhibit a significant HM+DNase-to-TF adjusted *R*^2^ ratio (i.e., demonstrating that HMs and DNase are more predictive of mRNA transcript abundance than TF binding). These processes and their respective ratios are listed in Additional file 4: Table S2. The specific TF and HM+DNase adjusted *R*^2^ values used to calculate the ratios for each of the 1,880 processes are provided in Additional file 5: Table S3.

**Figure 2:**
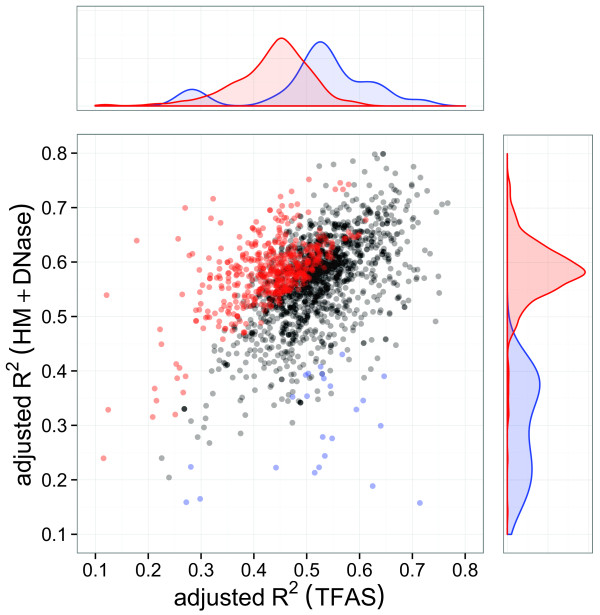
**Predictive power of TF binding and HM+DNase-based models.** These models are of mRNA transcript abundance for 1,880 sets of mESC genes grouped by ontology-classified biological processes. Sets of genes exhibiting significant HM+DNase-to-TF adjusted *R* ^2^ ratio (i.e., for which HMs are more predictive of transcript abundance) are indicated in red, with those exhibiting a significant TF-to-HM+DNase adjusted *R* ^2^ ratio (i.e., for which TF binding is more predictive) are indicated in blue. The overlap between the significant (Benjamini–Hochberg-corrected *P* < 0.05 [43]) and non-significant (grey) regions is due to the ratio significance threshold varying with the number of genes belonging to each group. HM, histone modification; TF, transcription factor; TFAS, transcription factor association strength.

The distributions of adjusted *R*^2^ values for models constructed from mESC TF and HM+DNase data (visualised in Figure 2) demonstrate that their relative predictive power is heterogeneous across different biological processes. A similar distribution of adjusted *R*^2^ values is evident for GM12878 data (not shown), although statistically significant outliers were not confidently identified due to an overall lower prediction accuracy of GM12878 models (described earlier). It is important to note that this lack of outliers does not adversely affect subsequent analysis, which focuses on statistically significant trends across high- and low-scoring biological processes rather than the individual processes themselves.

Our results suggest that the genome-wide redundancy reported in previous studies [15, 16] is not indicative of functional redundancy, but rather arises from averaging over heterogeneous groups of genes subject to different regulatory logic. Moreover, the observation that HM and DNase data were significantly more accurate in predicting the mRNA transcript abundance of mESC genes contributing to 28% of biological processes, suggests the existence of a small number of genes for which TF binding is considerably more informative. If under-represented in the processes examined, these genes would introduce negative bias into the null distribution (and therefore increase the number of statistically significant outliers) when randomly sampled during bootstrapped significance testing. This is also consistent with the order-of-magnitude fewer processes (1.3%) for which contributing mRNA transcript abundance was more accurately predicted by TF-binding data.

### Redundancy in transcriptional regulation is dependent upon enrichment for housekeeping genes

The set of genes for which TF binding is considerably more predictive of gene expression than HM and DNase data was comparatively small (Figure 2). Inspecting the biological processes with high TF-to-HM+DNase and HM+DNase-to-TF adjusted *R*^2^ ratios for their constituent genes, it is apparent that the former are enriched for housekeeping tasks (e.g., ncRNA processing and RNA splicing) and the latter for tissue and context-specific processes (e.g., signal transduction and regulation of cell differentiation). To investigate whether these two groups of genes can be characterised accordingly, the top 100 processes from each list were tested for enrichment of housekeeping genes.

**Figure 3:**
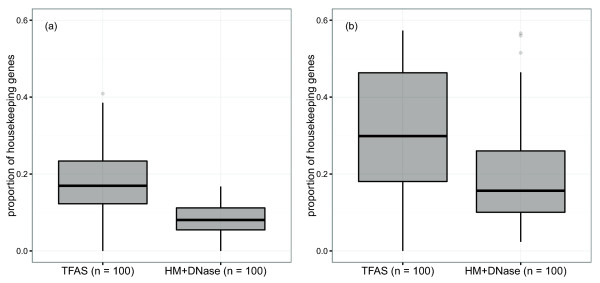
**Proportion of housekeeping genes contributing toward key biological processes.** These processes have the top 100 TF-to-HM+DNase (TF) and HM+DNase-to-TF (HM+DNase) adjusted *R* ^2^ ratios for **(a)** mESCs and **(b)** GM12878 cells. The proportion of housekeeping genes is significantly larger for the TF group in both cases (Welch’s *t*-test (a) *P* < 2.2 × 10^-16^ and (b) *P* < 2.6 × 10^-6^). This suggests that TF binding provides more information regarding the transcriptional regulatory state of mammalian biological processes enriched for housekeeping genes and conversely that HMs and DNase provide more information for tissue and context-sensitive processes. HM, histone modification; TF, transcription factor; TFAS, transcription factor association strength.

Figure 3 presents the housekeeping-gene enrichment of biological processes with the top 100 TF-to-HM+DNase and HM+DNase-to-TF adjusted *R*^2^ ratios for both (a) mESCs and (b) GM12878 cells. In both cases, the proportion of housekeeping genes contributing to biological processes is significantly larger for the top 100 TF-to-HM+DNase group (Welch’s *t*-test (a) *P* < 2.2 × 10^-16^ and (b) *P* < 2.6 × 10^-6^ [44]). These results suggest TF binding provides more information regarding the transcriptional regulatory state of mammalian biological processes enriched for housekeeping genes.

Our findings can be explained by considering the spatial chromatin structure, as housekeeping genes are known to maintain constant ubiquitous expression by co-location in regions of actively transcribed open chromatin (e.g., at the boundaries of euchromatin and heterochromatin, topologically associating domains and larger A and B compartments [6, 45, 46]). These regions are maintained primarily by the activity of boundary proteins (e.g., CTCF [47]) rather than histone acetylation and methyl-recognising co-factors [48]. They are also significantly depleted for histone H1 (responsible for solenoidal chromatin packing [49]) and exhibit overall nucleosomal sparsity to provide unrestricted TF accessibility [50]. As HM ChIP-seq data was not normalised to nucleosome density (not assayed), this combination of nucleosome sparsity and TF-modulated chromatin structure presumably explains why HMs provide comparatively little information regarding the regulatory state of housekeeping genes. As TF–DNA complexes in open regions are capable of remaining stable throughout multiple rounds of transcription [51, 52], it is also unsurprising that a snapshot of local TF binding would provide more information regarding housekeeping mRNA transcript abundance than in the case of dynamically modulated chromatin.

## Conclusions

Predictive modelling frameworks (recently reviewed in [14]) have the potential to fill an important gap between thermodynamically driven models of individual transcription regulatory events [53, 54] and association-driven network models of indirect gene regulation (e.g., those represented in the DREAM challenges [55]). Rather than modelling the regulation of specific genes, they can lead to more general conclusions regarding the roles and interactions of TFs, HMs and other key regulators of gene expression. Furthermore, they avoid the common issue of an underdetermined system by treating individual genes as *observations* of transcriptional regulatory logic in action, rather than *variables* in an association-driven analysis [56].

Recent predictive modelling studies have identified statistical redundancy between the regulatory roles of TFs and HMs [15, 16]. These findings outwardly contradict our understanding of transcriptional regulation, in which TFs and HMs play complementary yet distinct roles in RNAP-II recruitment and elongation. Moreover, there have previously been minimal attempts to resolve this contradiction (or even to distinguish between statistical and functional redundancy), potentially leading readers to perceive this modelling framework as one prone to capturing invalid biology. For the above reasons and to enhance our understanding of transcriptional regulatory logic, we believe that it is important to identify the underlying cause of this recurring statistical redundancy.

In this study, we validated the robustness of previous findings across multiple mammalian cell types and using different modelling algorithms. We extend this analysis by constructing individual models for thousands of ontology-classified biological processes, identifying significant variation in the relative predictive power of TFs and HMs across processes (i.e., the redundancy observed at the genome-wide level breaks down at this resolution). Importantly, this resolves the paradox between the distinct regulatory roles of TFs and HMs and the statistical redundancy within and between these elements.

Our investigation has highlighted several examples of simple predictive models yielding complex results that are consistent with our current understanding of fundamental molecular biology. With the hindsight provided by recent surveys of spatial genomic domains based on chromatin conformation capture [6], we can identify the signature of housekeeping genes localised to nucleosome-sparse domain boundaries by our inability to predict their expression using HM ChIP-seq data. Similarly, the statistical redundancy between TFs corresponds with the recently established notion of a transcription factor hierarchy, whereby the binding of a pioneer TF initiates a sequence of cooperative binding events that results in chromatin remodelling and/or RNAP-II recruitment [57]. The well-characterised crosstalk between TFs and HMs in regulating transcriptional initiation and elongation is also reflected in the statistical redundancy between TFs and HMs, and importantly our results have demonstrated that such correlation is unlikely to imply functionally redundant regulatory roles. These outcomes highlight the potential of predictive modelling as a powerful explorative framework for integrating heterogeneous genome-wide datasets to elucidate novel biology, and we encourage other researchers to incorporate such models in their own analysis pipelines.

## Methods

### Data availability and implementation

All *Homo sapiens* (GM12878 lymphoblastoid cell line) and *Mus musculus* (embryonic stem cell) data used in this study are detailed in Tables 2 and 3. All data and scripts are available online [58].

**Table 3:** ***Homo sapiens*** **(GM12878 lymphoblastoid cell line) data**

Data type	Data source	Notes
RNA-seq	ENCODE [59]	49,488 genesFPKM-normalised [60]
TSS	Ensembl hg19/GRCh37 [27]	Consider only most 5 ^′^-located TSS for each gene
TF ChIP-seq	ENCODE [59]	c-Fos, Ctcf, Egr1, Nrf1, Nrsf, Pou2f2, Sp1, Srf, Stat3, Usf1 and Yy1
HM ChIP-seq	ENCODE [59]	H3K4me1, H3K4me2, H3K4me3, H4K20me1, H3K27me3 and H3K36me3
DNase-seq	ENCODE [59]	DNase I hypersensitivity
Gene ontology	[33, 34]	GOC validation date: 21 March 2014 Structure from GO.db R package
Housekeeping annotations	[61, 62]	3,804 genes Using RNA-seq data GSE30611

Genes corresponding with haplotype variants, unmapped contig regions and low confidence RNA-seq mappings were removed, resulting in a set of 38,041 genes for analysis.

ChIP, chromatin immunoprecipitation; GOC, Gene Ontology Consortium; HM, histone modification; seq, sequencing; TF, transcription factor; TSS, transcription start site.

### Calculation of transcription factor–gene association strength

For each gene *i* and TF *j*, ChIP-seq binding data for *j* was integrated to calculate a *transcription factor association strength* (TFAS), *a*_*ij*_ [14, 15, 18]:
1

where *g*_*k*_ is the height (mapped tags) of the *k*th TF-binding peak, *d*_*k*_ is the distance (in base pairs) separating the *k*th peak from the TSS of gene *i*, and *d*_0_ is the empirical decay rate derived from the approximate average widths of ChIP-seq peaks (*d*_0_ = 5,000 for all TFs except E2f1 (*d*_0_ = 500) [14]). Binding sites further than 30,000 bp from the TSS were not considered as their weighted contribution is negligible. The TFAS matrix **A** was log-transformed and quantile-normalised [63].

An alternative formulation of the TFAS involves simply summing the number of mapped ChIP-seq tags either side of the TSS [16, 17] (e.g., -4 to approximately 4 kbp). The exponentially decaying formulation was chosen as it corresponds with the observed sharpness of ChIP-seq TF-binding peaks about the TSS and yields more accurate predictions of mRNA transcript abundance [16].

### Calculation of histone and DNase scores

For each pair of gene *i* and HM *j*, the number of mapped ChIP-seq tags for *j* was summed within a region 2,000 bp either side of the TSS of *i* to calculate a *histone score*, *b*_*ij*_ [14, 15]:
2

where *g*_*k*_ is the number of ChIP/DNase-seq reads for *j* mapped to position *k* relative to the TSS of *i*. A region 2,000 bp either side of the TSS was chosen for consistency with previous studies [15–17, 19]. An equivalent method was applied to DNase-seq tags to produce a *DNase score*. The concatenated histone and DNase score matrix **B** was log-transformed and quantile-normalised as per the TFAS matrix **A** [63]. Unlike TFs, HMs do not exhibit sharp, well-defined ChIP-seq peaks about the TSS. This prevents the formulation of histone and DNase scores equivalent to Equation 1 [16].

### Regression models for predicting mRNA transcript abundance

**Figure 4:**
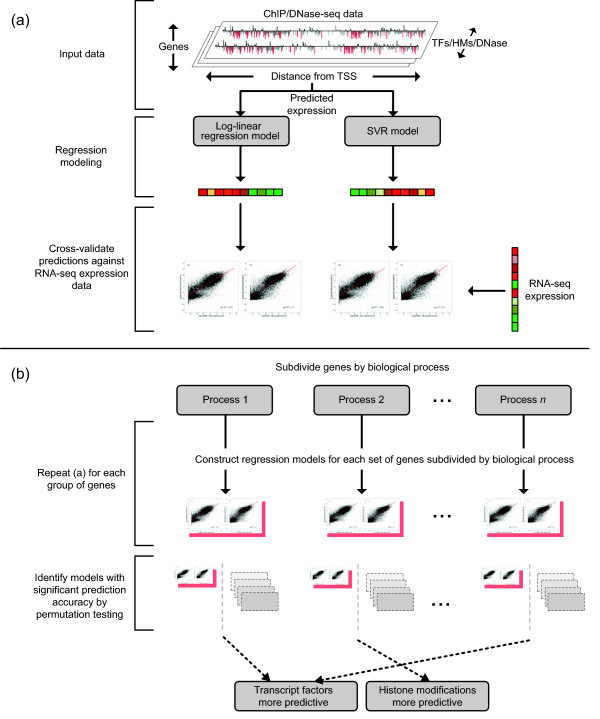
**Flowchart illustrating the experimental pipeline presented in this study.** ChIP/DNase-seq data were used to construct regression models of mRNA transcript abundance for a set of genes. The prediction accuracy of each model was evaluated relative to RNA-sequencing data. By constructing groups of genes categorised by biological process and applying the above methodology, it was possible to identify heterogeneity in the relative predictive power of TFs and HMs. These groups were later analysed for enrichment for housekeeping genes.

Predictive models of mRNA transcript abundance were constructed using two regression techniques: log-linear regression and SVR [64], as illustrated in Figure 4(a). Both techniques have been previously applied to modelling transcript abundance as a function of transcriptional regulatory elements and demonstrated to yield comparable predictive performance [15–19]. However, as log-linear regression and nonlinear SVR have previously been applied either to independent datasets or with different TFAS/histone score formulations, it remains unclear which is more appropriate for transcript abundance modelling.

#### Log-linear regression

The log-linear regression model describing mRNA transcript abundance as a function of TF binding, HMs and DNase was formulated as:
3

where *y*_*i*_ is the mRNA transcript abundance of gene *i*, *μ* is the basal transcript abundance and *ε*_*i*_ is the gene-specific error term [14, 15, 18]. For a gene *i* and TF *j*, *a*_*ij*_ is the TFAS defined in Equation 1 and *β*_*j*_ is the fitted coefficient. Similarly, *b*_*ik*_ is the histone (or DNase) score for a gene *i* and HM (or DNase) *k* defined in Equation 2 and *γ*_*k*_ is the fitted coefficient. The constant *σ* is fitted from a 20% held-aside dataset to avoid evaluation of log(0). A model considering only the TFAS or HM+DNase score data can be constructed by excluding the HM+DNase or TF-binding components from Equation 3, respectively. The linear regression implementation from the stats R package was used.

#### Support vector regression

The SVR model describing mRNA transcript abundance of a gene *i*, *y*_*i*_, as a nonlinear function of a TFAS and/or HM+DNase score matrix, **X**, can be formulated as:
4

where *K* (·) is the kernel function and ***α*** represents the difference between the Lagrange multipliers fitted using a constrained quadratic optimisation process (described in Additional file 6). The *ε*-SVR with radial basis kernel function implementation from the e1071 R package was used with default parameters.

### Identifying heterogeneity in predictive power

Previous models of mRNA transcript abundance have focused on modelling mRNA transcript abundance genome-wide [15–19]. To investigate whether the relative performance of TF-binding and HM+DNase-based models varies across smaller sets of process-related genes, the Gene Ontology biological process annotations for the available set of 17,517 mESC and 38,041 GM12878 genes were considered [34]. For each process, a set containing all genes annotated with that term or any descendant term was constructed. Sets containing fewer than 50 or greater than 10,000 genes were discarded, yielding 1,880 and 1,965 sets of genes (for mESC and GM12878 respectively) for analysis.

Two regression models were constructed for each set of genes: one considering only TF-binding data and the other considering only HM and DNase data. The performance of the fitted models was evaluated as an *adjustedR*^2^ score, which captures the proportion of variation in measured mRNA transcript abundance for those genes explained by the model. Unlike the *R*^2^ score (i.e., the *coefficient of determination*, equivalent to the square of the Pearson correlation coefficient and previously used to evaluate models of mRNA transcript abundance [16, 17]), the adjusted *R*^2^ prevents spurious inflation due to the introduction of additional explanatory variables [65]. The ratio of adjusted *R*^2^ values for the TF and HM+DNase models was calculated to capture their relative performance.

A bootstrapped non-parametric test was conducted to identify sets of genes exhibiting a significant adjusted *R*^2^ ratio, as illustrated in Figure 4(b). Specifically, for each biological process annotating *n* genes, 5,000 sets of *n* genes were randomly sampled from the available 17,517/38,041 (mESC/GM12878) to generate a corresponding distribution of adjusted *R*^2^ ratios under the null hypothesis. From this distribution, a non-parametric *P* value was calculated using an empirical cumulative distribution function approximation [66]. Statistically significant *P* values were identified by applying a Benjamini–Hochberg correction with a false discovery rate of 0.05 [43].

### Identifying enrichment of housekeeping genes

If the gene-specific residual, , for a gene, *i*, is sufficiently large, it follows that the relationship between mRNA transcript abundance and the transcriptional regulatory elements described by the corresponding regression model does not hold for *i*. These poorly fitted genes were removed to identify enrichment of housekeeping annotation amongst sets of genes sharing common regulatory profiles. For each of the biological processes subsequently found to exhibit a statistically significant adjusted *R*^2^ ratio for the TF and HM+DNase models, genes exhibiting a studentised residual magnitude  were therefore discarded [67]. The remaining genes were tested for significant enrichment of housekeeping annotation using the bootstrapped non-parametric test methodology described above.

## Electronic supplementary material

Additional file 1: Figure S1: Relationship between actual genome-wide mESC mRNA transcript abundance (RPKM-normalised RNA-sequencing) versus transcript abundance predicted from ChIP-seq (TF binding and HM) and DNase data. Two regression algorithms were considered: log-linear regression, for **(a)** TF, **(b)** HM+DNase and **(c)** TF+HM+DNase; and support vector regression, for **(d)** TF, **(e)** HM+DNase and **(f)** TF+HM+DNase. ChIP-seq, chromatin immunoprecipitation sequencing; HM, histone modification; mESC, mouse embryonic stem cell; TF, transcription factor. (ZIP 11 MB)

Additional file 2: Figure S2: Relationship between actual genome-wide GM12878 mRNA transcript abundance (FPKM-normalised RNA-sequencing) versus transcript abundance predicted from ChIP-seq (TF binding and HM) and DNase data. Two regression algorithms were considered: log-linear regression, for **(a)** TF, **(b)** HM+DNase and **(c)** TF+HM+DNase; and support vector regression, for **(d)** TF, **(e)** HM+DNase and **(f)** TF+HM+DNase. ChIP-seq, chromatin immunoprecipitation sequencing; HM, histone modification; TF, transcription factor. (ZIP 19 MB)

Additional file 3: Table S1: List of 25 mESC biological processes found to exhibit a statistically significant TF-to-HM+DNase adjusted *R* ^2^ ratio (demonstrating that TF binding is more predictive of mESC mRNA transcript abundance than HMs and DNase for the markers considered in this study, Benjamini–Hochberg-corrected *P* < 0.05 [43]). (CSV 1 KB)

Additional file 4: Table S2: List of 523 mESC biological processes found to exhibit a statistically significant HM+DNase-to-TF adjusted *R* ^2^ ratio (i.e., demonstrating that HMs and DNase are more predictive of mESC mRNA transcript abundance than TF binding for the markers considered in this study, Benjamini–Hochberg-corrected *P* < 0.05 [43]). (CSV 30 KB)

Additional file 5: Table S3: TF and HM+DNase model performance for each of the 1,880 mESC biological processes considered. (CSV 125 KB)

Additional file 6: **The** ***ε*** **-support vector regression model describing mRNA transcript abundance as a nonlinear function of a TFAS or HM+DNase score matrix.** (PDF 37 KB)

## Abbreviations

bp: base pair
ChIP: chromatin immunoprecipitation
HM: histone modification
kbp: kilobase pair
mESC: mouse embryonic stem cell
RNAP-II: RNA polymerase II
SVR: support vector regression
TF: transcription factor
TFAS: transcription factor association strength
TSS: transcription start site.

## References

[1] Maston GA, Evans SK, Green MR (2006). Transcriptional regulatory elements in the human genome. Annu Rev Genomics Hum Genet.

[2] Farnham PJ (2009). Insights from genomic profiling of transcription factors. Nat Rev Genet.

[3] Vaquerizas JM, Kummerfeld SK, Teichmann SA, Luscombe NM (2009). A census of human transcription factors: function, expression and evolution. Nat Rev Genet.

[4] Berger SL (2007). The complex language of chromatin regulation during transcription. Nature.

[5] Kurdistani SK, Tavazoie S, Grunstein M (2004). Mapping global histone acetylation patterns to gene expression. Cell.

[6] Dekker J, Marti-Renom MA, Mirny LA (2013). Exploring the three-dimensional organization of genomes: interpreting chromatin interaction data. Nat Rev Genet.

[7] Li B, Carey M, Workman JL (2007). The role of chromatin during transcription. Cell.

[8] Bernstein BE, Meissner A, Lander ES (2007). The mammalian epigenome. Cell.

[9] Pekowska A, Benoukraf T, Ferrier P, Spicuglia S (2010). A unique H3K4me2 profile marks tissue-specific gene regulation. Genome Res.

[10] Kouzarides T (2007). Chromatin modifications and their function. Cell.

[11] Krivtsov AV, Feng Z, Lemieux ME, Faber J, Vempati S, Sinha AU, Xia X, Jesneck J, Bracken AP, Silverman LB, Kutok JL, Kung AL, Armstrong SA (2008). H3K79 methylation profiles define murine and human MLL-AF4 leukemias. Cancer Cell.

[12] Shi Y (2007). Histone lysine demethylases: emerging roles in development, physiology and disease. Nat Rev Genet.

[13] Portela A, Esteller M (2010). Epigenetic modifications and human disease. Nat Biotechnol.

[14] Budden DM, Hurley DG, Crampin EJ (2014). **Predictive modelling of gene expression from transcriptional regulatory elements**. Brief Bioinform.

[15] McLeay RC, Lesluyes T, Partida GC, Bailey TL (2012). Genome-wide in silico prediction of gene expression. Bioinformatics.

[16] Cheng C, Gerstein M (2012). Modeling the relative relationship of transcription factor binding and histone modifications to gene expression levels in mouse embryonic stem cells. Nucleic Acids Res.

[17] Cheng C, Yan K-K, Yip KY, Rozowsky J, Alexander R, Shou C, Gerstein M (2011). A statistical framework for modeling gene expression using chromatin features and application to modENCODE datasets. Genome Biol.

[18] Ouyang Z, Zhou Q, Wong WH (2009). ChIP-Seq of transcription factors predicts absolute and differential gene expression in embryonic stem cells. Proc Natl Acad Sci.

[19] Karlić R, Chung H-R, Lasserre J, Vlahoviček K, Vingron M (2010). Histone modification levels are predictive for gene expression. Proc Natl Acad Sci.

[20] Arlot S, Celisse A (2010). A survey of cross-validation procedures for model selection. Stat Surv.

[21] Chen X, Xu H, Yuan P, Fang F, Huss M, Vega VB, Wong E, Orlov Y. L, Zhang W, Jiang J, Loh Y-H, Yeo HC, Yeo ZX, Narang V, Govindarajan KR, Leong B, Shahab A, Ruan Y, Bourque G, Sung W-K, Clarke ND, Wei C-L, Ng H-H (2008). Integration of external signaling pathways with the core transcriptional network in embryonic stem cells. Cell.

[22] Meissner A (2010). Epigenetic modifications in pluripotent and differentiated cells. Nat Biotechnol.

[23] Epsztejn-Litman S, Feldman N, Abu-Remaileh M, Shufaro Y, Gerson A, Ueda J, Deplus R, Fuks F, Shinkai Y, Cedar H, Bergman Y (2008). De novo DNA methylation promoted by G9a prevents reprogramming of embryonically silenced genes. Nat Struct Mol Biol.

[24] Feldman N, Gerson A, Fang J, Li E, Zhang Y, Shinkai Y, Cedar H, Bergman Y (2006). G9a-mediated irreversible epigenetic inactivation of Oct-3/4 during early embryogenesis. Nat Cell Biol.

[25] Cloonan N, Forrest AR, Kolle G, Gardiner BB, Faulkner GJ, Brown MK, Taylor DF, Steptoe AL, Wani S, Bethel G, Robertson AJ, Perkins AC, Bruce SJ, Lee CC, Ranade SS, Peckham HE, Manning JM, McKernan KJ, Grimmond SM (2008). Stem cell transcriptome profiling via massive-scale mRNA sequencing. Nat Methods.

[26] Mortazavi A, Williams BA, McCue K, Schaeffer L, Wold B (2008). Mapping and quantifying mammalian transcriptomes by RNA-Seq. Nat Methods.

[27] Flicek P, Amode MR, Barrell D, Beal K, Billis K, Brent S, Carvalho-Silva D, Clapham P, Coates G, Fitzgerald S, Gil L, Gironi CG, Gordon L, Hourlier T, Hunt S, Johnson N, Juettemann T, Kahari AK, Keenan S, Kulesha E, Martin FJ, Maurel T, McLaren WM, Murphy DN, Nag R, Overduin B, Pignatelli M, Pritchard B, Pritchard E, Riat HS (2014). Ensembl 2014. Nucleic Acids Res.

[28] **Mapping of transcription factor binding sites in mouse embryonic stem cells**. [http://www.ncbi.nlm.nih.gov/geo/query/acc.cgi?acc=GSE11431]

[29] **MIT CHiP-seq data**. [ftp://ftp.broad.mit.edu/pub/papers/chipseq/]

[30] Mikkelsen TS, Ku M, Jaffe DB, Issac B, Lieberman E, Giannoukos G, Alvarez P, Brockman W, Kim T-K, Koche RP, Lee W, Mendenhall E, O’Donovan A, Presser A, Russ C, Xie X, Meissner A, Wernig M, Jaenisch R, Nusbaum C, Lander ES, Bernstein BE (2007). Genome-wide maps of chromatin state in pluripotent and lineage-committed cells. Nature.

[31] Meissner A, Mikkelsen TS, Gu H, Wernig M, Hanna J, Sivachenko A, Zhang X, Bernstein BE, Nusbaum C, Jaffe DB, Gnirke A, Jaenisch R, Lander ES (2008). Genome-scale DNA methylation maps of pluripotent and differentiated cells. Nature.

[32] **Genome-wide in silico prediction of gene expression**. [http://research.imb.uq.edu.au/t.bailey/supplementary_data/McLeay2011a/] 10.1093/bioinformatics/bts529 PMC3476338 22954627

[33] **Gene Ontology Consortium download annotations**. [http://www.geneontology.org/GO.downloads.annotations.shtml]

[34] Ashburner M, Ball CA, Blake JA, Botstein D, Butler H, Cherry JM, Davis AP, Dolinski K, Dwight SS, Eppig JT, Harris MA, Hill DP, Issel-Tarver L, Kasarskis A, Lewis S, Matese JC, Richardson JE, Ringwald M, Rubin GM, Sherlock G (2000). Gene ontology: tool for the unification of biology. Nat Genet.

[35] **Mouse Genome Informatics**. [http://www.informatics.jax.org/homology.shtml]

[36] Voigt P, Tee W-W, Reinberg D (2013). A double take on bivalent promoters. Genes Dev.

[37] Hu G, Cui K, Northrup D, Liu C, Wang C, Tang Q, Ge K, Levens D, Crane-Robinson C, Zhao K (2013). H2A.Z facilitates access of active and repressive complexes to chromatin in embryonic stem cell self-renewal and differentiation. Cell Stem Cell.

[38] Ku M, Jaffe JD, Koche RP, Rheinbay E, Endoh M, Koseki H, Carr SA, Bernstein BE (2012). H2A.Z landscapes and dual modifications in pluripotent and multipotent stem cells underlie complex genome regulatory functions. Genome Biol.

[39] Jin VX, O’Geen H, Iyengar S (2007). Identification of an OCT4 and SRY regulatory module using integrated computational and experimental genomics approaches. Genome Res.

[40] Xu X, Bieda M, Jin VX, Rabinovich A, Oberley MJ, Green R, Farnham PJ (2007). A comprehensive ChIP–chip analysis of E2F1, E2F4, and E2F6 in normal and tumor cells reveals interchangeable roles of E2F family members. Genome Res.

[41] Schreiber SL, Bernstein BE (2002). Signaling network model of chromatin. Cell.

[42] Baba K, Shibata R, Sibuya M (2004). Partial correlation and conditional correlation as measures of conditional independence. Aust N Z J Stat.

[43] Benjamini Y, Hochberg Y (1995). Controlling the false discovery rate: a practical and powerful approach to multiple testing. J R Stat Soc Series B (Methodological).

[44] Welch BL (1947). The generalization of Student’s problem when several different population variances are involved. Biometrika.

[45] Dixon JR, Selvaraj S, Yue F, Kim A, Li Y, Shen Y, Hu M, Liu JS, Ren B (2012). Topological domains in mammalian genomes identified by analysis of chromatin interactions. Nature.

[46] Zhou G-L, Xin L, Song W, Di L-J, Liu G, Wu X-S, Liu D-P, Liang C-C (2006). Active chromatin hub of the mouse *α*-globin locus forms in a transcription factory of clustered housekeeping genes. Mol Cell Biol.

[47] Phillips JE, Corces VG (2009). CTCF: Master weaver of the genome. Cell.

[48] Gaszner M, Felsenfeld G (2006). Insulators: exploiting transcriptional and epigenetic mechanisms. Nat Rev Genet.

[49] Thoma F, Koller T, Klug A (1979). Involvement of histone H1 in the organization of the nucleosome and of the salt-dependent superstructures of chromatin. J Cell Biol.

[50] Tazi J, Bird A (1990). Alternative chromatin structure at CpG islands. Cell.

[51] Cloutier TE, Librizzi MD, Mollah A, Brenowitz M, Willis IM (2001). Kinetic trapping of DNA by transcription factor IIIB. Proc Natl Acad Sci.

[52] Nikolov D, Burley S (1997). RNA polymerase II transcription initiation: a structural view. Proc Natl Acad Sci.

[53] He X, Samee MAH, Blatti C, Sinha S (2010). Thermodynamics-based models of transcriptional regulation by enhancers: the roles of synergistic activation, cooperative binding and short-range repression. PLoS Comput Biol.

[54] Mariani L, Löhning M, Radbruch A, Höfer T (2004). Transcriptional control networks of cell differentiation: insights from helper T lymphocytes. Progress Biophys Mol Biol.

[55] Marbach D, Costello JC, Küffner R, Vega NM, Prill RJ, Camacho DM, Allison KR, Kellis M, Collins JJ, Stolovitzky G (2012). Wisdom of crowds for robust gene network inference. Nat Methods.

[56] Maetschke SR, Madhamshettiwar PB, Davis MJ, Ragan MA (2014). Supervised, semi-supervised and unsupervised inference of gene regulatory networks. Brief Bioinform.

[57] Sherwood RI, Hashimoto T, O’Donnell CW, Lewis S, Barkal AA, van Hoff JP, Karun V, Jaakkola T, Gifford DK (2014). Discovery of directional and nondirectional pioneer transcription factors by modeling DNase profile magnitude and shape. Nat Biotechnol.

[58] **Scripts and supplementary data**. [http://sourceforge.net/projects/budden2014exploring/]

[59] The ENCODE Project Consortium (2012). An integrated encyclopedia of DNA elements in the human genome. Nature.

[60] Trapnell C, Williams BA, Pertea G, Mortazavi A, Kwan G, van Baren MJ, Salzberg SL, Wold BJ, Pachter L (2010). Transcript assembly and quantification by RNA-Seq reveals unannotated transcripts and isoform switching during cell differentiation. Nat Biotechnol.

[61] **List of housekeeping genes**. [http://www.tau.ac.il/elieis/HKG/]

[62] Eisenberg E, Levanon EY (2013). Human housekeeping genes, revisited. Trends Genet.

[63] Bolstad BM, Irizarry RA, Åstrand M, Speed TP (2003). A comparison of normalization methods for high density oligonucleotide array data based on variance and bias. Bioinformatics.

[64] Basak D, Pal S, Patranabis DC (2007). Support vector regression. Neural Inf Processing Letters Rev.

[65] Harel O (2009). The estimation of *R*^2^ and adjusted *R*^2^ in incomplete data sets using multiple imputation. J Appl Stat.

[66] Knijnenburg TA, Wessels LF, Reinders MJ, Shmulevich I (2009). Fewer permutations, more accurate *p*-values. Bioinformatics.

[67] Wang C, Tian R, Zhao Q, Xu H, Meyer CA, Li C, Zhang Y, Liu XS (2012). Computational inference of mRNA stability from histone modification and transcriptome profiles. Nucleic Acids Res.

