# Supplementary material for: Predicting expression: the complementary power of histone modification and transcription factor binding data
Source: Epigenetics Chromatin. 2014 Nov 24;7:36. doi: 10.1186/1756-8935-7-36 (PMC4258808; doi:10.1186/1756-8935-7-36)
Supplement: Supplementary file 6 — Additional file 6: The ε -support vector regression model describing mRNA transcript abundance as a nonlinear function of a TFAS or HM+DNase score matrix. (PDF 37 KB) [file 13072_2014_340_MOESM6_ESM.pdf]

Supplementary Method 1

The ϵ-support vector regression (ϵ-SVR) model describing mRNA transcript abundance of a gene i, y_i, as a nonlinear function of a TFAS or HM+DNase score matrix, $\mathbf{X}$, can be formulated as [1]:

$$y_i = \mu + \sum_i (\alpha_i^* - \alpha_i) K(X_i, \boldsymbol{X}) + \varepsilon_i,$$

where the kernel function, $K(X_i, \mathbf{X}) = \Phi(X_i) \cdot \Phi(\mathbf{X})$, implicitly maps $\mathbf{X} \mapsto \Phi(\mathbf{X})$ to allow a nonlinear relationship, $Y = f(\mathbf{X})$, to be projected from a linear relationship in the higher-dimensional mapped space, Φ (a process known as the "kernel trick"). The optimal Lagrange multipliers, $\boldsymbol{\alpha}$, can be determined by solving the following constrained, quadratic optimisation problem:

$$\min_{\boldsymbol{\alpha}, \boldsymbol{\alpha}^*} \left\{ \begin{array}{l} \dfrac{(\boldsymbol{\alpha} - \boldsymbol{\alpha}^*)^\top Q (\boldsymbol{\alpha} - \boldsymbol{\alpha}^*)}{2} \\ + \epsilon \sum_i (\alpha_i + \alpha_i^*) + \epsilon \sum_i (\alpha_i - \alpha_i^*) \end{array} \right.$$

$$\text{subject to} \left\{ \begin{array}{l} \mathbf{1}^\top (\boldsymbol{\alpha} - \boldsymbol{\alpha}^*) = 0 \\ 0 \leq \alpha_i, \alpha_i^* \leq C \quad i = 1, \ldots, n \end{array} \right.$$

where $C > 0$ is the regularisation parameter, $\epsilon > 0$ controls the width of the ϵ-insensitive loss region [2] and $Q_{ij} = K(X_i, X_j)$. The radial basis function (RBF) kernel, $K(X_i, X_j) = \exp\left(-\gamma ||X_i - X_j||^2\right)$, was applied.

References

[1] Drucker, H., Burges, C. J., Kaufman, L., Smola, A., and Vapnik, V. (1997) Support vector regression machines *Advances in neural information processing systems* **9**, 155–161.

[2] Vapnik, V. N. (1995) The Nature of Statistical Learning Theory, Springer, .
